# Classroom-based physical activity improves children’s math achievement – A randomized controlled trial

**DOI:** 10.1371/journal.pone.0208787

**Published:** 2018-12-17

**Authors:** Mona Have, Jacob Have Nielsen, Martin Thomsen Ernst, Anne Kaer Gejl, Kjeld Fredens, Anders Grøntved, Peter Lund Kristensen

**Affiliations:** 1 Centre of Research in Childhood Health, Department of Sports Science and Clinical Biomechanics, University of Southern, Odense, Denmark; 2 Department of Learning and Philosophy, Aalborg University, Aalborg, Denmark; University of New Mexico, UNITED STATES

## Abstract

This RCT investigated the effect on children of integrating physical activity (PA) into math lessons. The primary outcome was math achievement and the secondary outcomes were executive functions, fitness and body mass index. Twelve Danish schools were randomized to either an intervention group or a control group. A total of 505 children with mean age 7.2 ± 0.3 years were enrolled in the study. Change in math achievement was measured by a 45-minute standardized math test, change in executive function by a modified Eriksen flanker task, aerobic fitness by the Andersen intermittent shuttle-run test, and body mass index by standard procedures. PA during the math lessons and total PA (including time spent outside school) were assessed using accelerometry (ActiGraph, GT3X and GT3X+). Children in the intervention group improved their math score by 1.2 (95% CI 0.3; 2.1) more than the control group (p = 0.011) and had a tendency towards a higher change in physical activity level during math lessons of 120,4 counts/min (95% CI -9.0;249.8.2, p = 0.067). However, the intervention did not affect executive functions, fitness or body mass index. Participation in a 9-month PA intervention (from 2012–2013) improved math achievement among elementary school children. If replicated, these findings would suggest that implementation of physical activity in school settings could lead to higher academic achievement.

## Introduction

A goal of any education system is how to optimize educational programs so that children can learn the academic skills that will enable them to improve the future for their societies and families. The crucial question is which initiatives might be effective in stimulating the learning process. The growing demands for improved quality in education, along with reduced financial resources, are often challenging for schools, especially in western societies. An emphasis on standardized testing has led to one strategy where lower priority is given to physical education in favor of spending more time on academic instruction [1], thus considering that the physical aspects of the human body are irrelevant to cognition and the acquisition of knowledge [2]. However, a number of cognitive and physiological benefits have been associated with physical activity (PA) [3, 4], suggesting that reduction of PA may be harmful to children’s academic achievement.

Physical activity appears to have both immediate effects on cognitive functions [5–7] and more long-term effects when people undertake regular exercise e.g. [8–11]. However, only a few high-quality longitudinal studies have examined the effect of PA on the academic performance of schoolchildren [12, 13]. A study conducted over three years among US elementary school children (2^nd^-3^rd^ to 4^th^-5^th^ grade) showed greater academic improvement in the group receiving physically active academic lessons compared to controls [12]. However, since the main objective was to reduce body mass index through PA, the academic results were considered secondary and were based on a subsample (n = 203) of the study. Mullender-Wijnsma and co-workers recently reported that physically active academic lessons over two years (22 weeks per year) significantly improved the math and spelling performance of 8-year-old schoolchildren [13]. Other studies have reported no or little academic improvement compared to controls after a PA intervention [14–17]. These discrepancies may be due to relatively short intervention periods e.g. one day [14, 18], 28 days [17], and three months [16] as well as the lack of pre-intervention measurements [14, 16]. This research area thus needs large, long-term studies that focus on investigating the effects of classroom-based PA on academic achievement.

We designed a randomized controlled trial with the primary objective of investigating how math achievement was affected by task-relevant physical activity incorporated into math teaching for 7-year-old schoolchildren. We define task-relevant physical activity as specific physical activity that enables or supports learning due to a meaningful connection between the activity and the learning task while task-irrelevant physical activity here is defined as whole-body activities that are not integrated into the learning task. Secondary aims were to investigate the effects on executive functions, aerobic fitness, and body mass index.

## Material and methods

**Fig 1:**
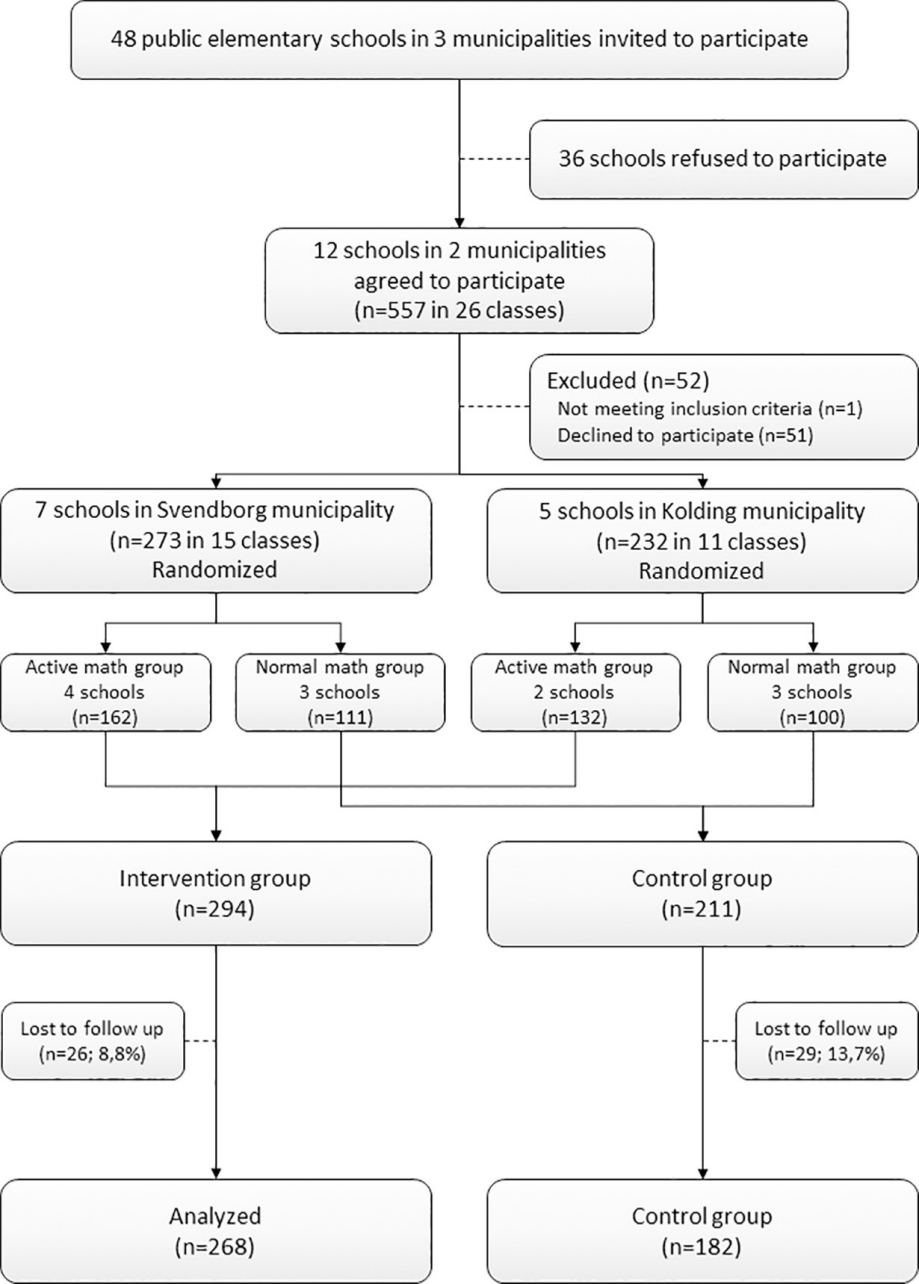
Flowchart of the recruitment and randomization process.

Study design and methods have previously been described in greater detail [19]. In short, the study was designed as a cluster-randomized controlled trial involving 1^st^ grade children in 12 schools from two municipalities in the Region of Southern Denmark (Fig 1). Randomization was performed by random selection of sealed envelopes containing the intervention allocation stratified by municipality, in the presence of school leaders, municipality representatives and study researchers. Participants were recruited in May and June 2012. Of the 557 children invited to participate, 90.7% (n = 505) met the inclusion criteria of having no physical disability and giving written parental consent. Because the two municipalities differed in the number of weekly physical education classes during school time, the study subjects were randomized into intervention or control group at school level, stratified by municipality. Children in Svendborg municipality received six lessons per week (270 minutes), and children in Kolding received two lessons per week (90 min). One out of three municipalities declined due to lack of time resources. We found no significant differences between the two municipalities in age profile, inclusion in the workforce or socioeconomic status (data from Statistics Denmark). The trial protocol was approved by the ethics committee of the Region of Southern Denmark (S-20140105) (S2 File) and registered at clinicaltrials.gov (NCT02488460).

The intervention group received classroom-based PA incorporated into math lessons for one school year. Subjects in the intervention group received an average of 6 math lessons of 45 minutes per week during the intervention. Each 45-minute lesson consisted of at least 15 minutes of PA spread over the lesson, and sedentary activities were limited to bouts of maximum 20 minutes. Subjects in the control group received regular classroom instruction, also with an average of 6 math lessons of 45 minutes per week.

Intervention group teachers attended a mandatory training course consisting of four workshops including two workshops two-three weeks prior to the start of the intervention, and two follow-up workshops during the first four months of the intervention. The aim of the course was to provide teachers with the skills to implement task-relevant physical activity into the math teaching, i.e. to use suggested methods and develop new ways of integrating physical activity into lessons as well as organizing activities. Furthermore, the course aimed at providing understanding on how to report fidelity assessments. (for an overview of the course plan, see Have et al. 2016 [19]). Training course material was developed by the project leader and included specific instructions and inspiration for how teachers could integrate PA as an element of academic instruction in math lessons. Examples from this material were used to help illustrate the practical dimensions of the active math lessons. The material was focused on developing the teacher’s own abilities to design active math lessons and thus their own pedagogical skills and knowledge rather than handing out a complete manual with activities for the math lessons. The course included an introduction to the study context and aims, with emphasis on creating high motivation among the teachers for the intervention by explaining the potential positive cognitive effects of learning through PA and describing previous findings related to PA in general. At the last workshop, active teaching techniques were discussed and related to motivational, organizational, and management techniques. Several teachers attending the course highlighted the challenges they expected to face when implementing active math. This initiated an internet-based intra-school communication forum in each of the two municipalities, where the teachers shared activities, experiences, and advice for the active math intervention. The teachers received normal salary for attending the course as compensation for their time.

One example of an active math activity in the curriculum of the mandatory training course is Skipping rope. In this activity, the children are divided into pairs and they skip numbers or calculations to each other. One child skips while the other counts. If the child skips on two legs, one skip equals 10, and a one-legged skip equals 1. Thus, if a child skips three times with both legs and two times with one leg, the answer will be 32. Calculations can be made with rules of ‘addition’, by skipping forward and ‘subtraction’, by skipping backwards as well as ‘multiplication’ by skipping once with arms crossed. This activity addresses understanding of numbers and quantities, with higher quantities requiring more skips providing a bodily experience of more jumps with larger numbers. Furthermore, understanding of a simple multiplication table is addressed with the possibility of skipping tens with two legs, coupled with sensory cues of uni- and bipedal skipping. Finally understanding of mathematical concepts is integrated in terms of addition, subtraction and multiplication linked to forward (positive), backward (negative) and crossed skipping. With this addition a congruent sensorimotor input is provided with forward skipping being related to increase in numbers, moving forward on the number line, and backward skipping being related to decrease in numbers moving backwards on the number line.

Intervention compliance was encouraged through personal meetings with the math teachers as well as Short Message Service (SMS) questionnaires distributed every day for one week each month. These assessed teacher motivation for implementing PA as well as type and frequency of the PA implemented.

### Baseline and follow-up assessments

Assessment of math skills, executive functions, aerobic fitness, body composition and physical activity level was performed prior to start up in August 2012 and again immediately before the end of the intervention in June 2013. Math achievement was expressed by scores in a standardized math test, and secondary outcomes were executive function, fitness, and BMI. The scoring of all tests was blinded to the investigators.

#### Primary outcome: Math skills test

A detailed description of applied tests has previously been published [19]. Math skills were evaluated using a 45-minute standardized math test (MG) that was specifically designed for this age group by the developer of the Danish national tests (Hoegrefe Forlag) [20]. It consisted of 24 tasks assessing calculus and math in terms of the understanding of quantity and numbers, relations, addition, subtraction and geometry, with the final score ranging between 0 and 24. This type of test has been used in Danish primary schools for more than 25 years. It is based on multiple-choice questions, and the different response options were selected in such a way that incorrect options related to typical mistakes made by students in 1st grade. The test provides a score reflecting the participant’s math skills and was conducted individually with paper and pencil in a classroom, with no aids permitted. Following a general presentation of the test, the participants were presented with each task separately and given 1 min to answer. The number of correct answers was used for further analysis.

#### Secondary outcomes

*Executive functions* were evaluated using a computer-based modified Erickson Flanker task [21]. For a more detailed description of the Flanker task, see [19]. The response time of the different tasks were recorded and the following variables were calculated: i) % accurate congruent answers, ii) % accurate incongruent answers, iii) the reaction time for correct congruent answers, and iv) the reaction time for correct incongruent answers.

*Aerobic fitness* was assessed using the Andersen test [22]. The children were instructed to run as far as possible in 10 minutes back and forth between two lines 20 m apart. The test score was total distance in meters run by each child.

Height was measured without shoes to the nearest 0.5 cm using a Harpenden stadiometer (West Sussex, UK). Weight was measured in light clothing to the nearest 0.1 kg using an electronic scale (Tanita BWB-800, Tokyo, Japan). *Body mass index* was calculated as body mass (kg) divided by height (m) squared.

#### Assessment of classroom physical activity and total physical activity

The level of PA during math lessons and total daily PA were assessed using accelerometry (ActiGraph, GT3X and GT3X+, ActiGraph LLC, Pensacola, FL, USA). For a more detailed description, see [19]. PA data were collected for eight days at baseline and again just before the other follow-up measurements. A valid measurement of total PA was defined as a minimum of four days with at least 10 hours of recorded activity each day. Propero software (University of Southern Denmark, Odense, Denmark) was used to prepare the data for further analysis and to specify the time-points at which math lessons took place according to the class schedule. Total PA was expressed as mean counts per minute and as mean daily minutes in moderate-to-vigorous physical activity (defined using cut-off points by Evenson et al. [23]).

#### Effect of general vs. task-relevant physical activity stimulus

The difference between municipalities in normal physical education (PE) stimulus (270 vs. 90 min) provided an opportunity to investigate the impact of the PA stimulus as a general (extra PE) versus a task-relevant (active math) stimulus. Post-hoc analysis was conducted to examine the effect of the intervention on math achievement in four sub-groups: i) control (n = 100): no active math and 90 min PE per week (normal Danish standard), ii) extra PE (n = 111): no active math and 270 min PE per week, iii) active math (n = 132): active math and 90 min PE per week, iv) active math and extra PE (n = 162): active math and 270 min PE per week.

### Sample size and power

Based on data on standard deviation of the change in math score (3.6 points) and an estimated school-level intraclass correlation (0.07) obtained post hoc, the estimated minimal detectable difference in the change in math score between the intervention and control group was 1.9 points with a 2-sided 5% alpha level and 80% power.

### Statistics

Differences in baseline characteristics between the intervention and control groups were tested using Student´s independent t-tests, chi-square test, and the Wilcoxon rank sum test in case of skewed data. The effect of the intervention was tested using mixed effects regression models adjusted for gender, allocation group and baseline values of the dependent variable. Because of the clustered nature of the data, schools were included as random effects in the analyses and the Kenward-Roger degrees of freedom approximation was used. In order to test for effect modification by gender, an interaction term for gender with allocation group was included in the analyses.

To examine the impact of attrition, two sensitivity analyses were performed where missing data were imputed at follow-up for the main outcome using cluster mean imputation and group mean imputation, respectively, as described by Taljaard et al. [24]. For the group mean imputation, the values were predicted from a regression model using only control group data to give a conservative estimate of the effect size.

Relative Cohen’s d was calculated to provide a standardized measure of effect-size for group differences. In order to compare with the literature, absolute effect-size relative to baseline was calculated for intervention and control groups as well. Analyses were performed with statistical software STATA version 14.

## Results

The mean age of the 505 children who participated in the study was 7.2 ± 0.3 years. There were no significant differences at baseline between intervention and control group in any descriptive characteristics except height, with the control group significantly taller than the intervention group (p<0.001) (Table 1).

**Table 1:** Baseline characteristics of the study population.

	Control		Intervention	
**n**	211		294	
**Age** (years)	7.2	(0.3)	7.2	(0.3)
**Gender** (% boys)	51.7		48.0	
**Height** (m)	127.9	(5.2)	125.9	(5.7)
**Weight** (kg)	25.9	(5.1)	25.6	(4.1)
**BMI** (kg/m^2^)	15.7	(2.2)	16.1	(1.7)
**Math score** (points) *	18	(14;20)	18	(15;20)
**Total physical activity** (count/min)	658.2	(169.2)	657.9	(151.5)
**Total time at MVPA**^**a**^ (min/day)	75.2	(1.9)	77.0	(1.5)
**Physical activity in math class** (count/min)	550.0	(277.9)	403.7	(181.6)
**Accelerometer wear time** (hours/day)	12.9	(0.05)	12.9	(0.04)
**Accelerometer accepted days** (n)	6.1	(0.1)	6.4	(0.1)
**Cardiorespiratory fitness** (m)	848.6	(106.6)	854.1	(102.6)
**Congruent reaction time** (ms)	1390.1	(358.6)	1364.0	(353.0)
**Incongruent reaction time** (ms)	1742.3	(452.2)	1758.5	(416.6)
**Congruent accuracy** (%)	93.3	(13.4)	94.4	(10.4)
**Incongruent accuracy** (%)	76.1	(16.8)	76.4	(15.9)

Data are presented as mean (SD), or percentage.

* Denotes non-normal distributed data with medians and percentiles (25th, 75th) presented.

^a^ extrapolated to a 14 hours day length.

During the 9-month intervention period, the dropout rate was 13.7% in the control group and 8.8% in the intervention group, which was not statistically significant. Dropouts were mainly attributed to subjects not present at follow-up trials due to sickness or moving to a different city as well as subjects not being able to complete the test due to injury (e.g. the fitness test). As shown in Table 2, we observed no significant differences in any characteristics between children who completed the study and children who had missing data or were lost to follow-up. Furthermore, the drop-out rate was not significantly related to gender or baseline math score.

**Table 2:** Comparison of baseline characteristics of participants followed up at 9 months and those lost to follow-up or with missing data.

	Followed up		Lost or missing data at follow up		Diff. (95% CI)				p value
**Group allocation** (% in control group)	40.4		52.7		12.3				0.08
**Age** (years)	7.2	(n = 450)	7.2	(n = 45)	-0.05	(-0.2	;	0.05)	0.30
**Gender** (% boys)	50.0	(n = 450)	45.5	(n = 55)	-4.4	*			0.52
**Height** (m)	126.8	(n = 442)	125.8	(n = 43)	-0.93	(-2.7	;	0.8)	0.29
**Weight** (kg)	25.8	(n = 442)	25.1	(n = 43)	0.70	(-2.1	;	0.7)	0.33
**BMI** (kg/m^2^)	15.9	(n = 442)	15.7	(n = 43)	-0.24	(-0.8	;	0.4)	0.45
**Math score** (points)	18	(n = 450)	16	(n = 37)	2.0	*			0.10
**Total physical activity** (count/min)	658.7	(n = 362)	650.0	(n = 28)	-8.6	(-69.9	;	52.7)	0.78
**Physical activity in math class** (count/min)	423.2	(n = 403)	449.6	(n = 38)	26.4	*			0.27
**Cardiorespiratory fitness** (m)	852.9	(n = 430)	840.6	(n = 38)	-12.3	(-46.9	;	22.4)	0.49
**Congruent reaction time** (ms)	1373.8	(n = 436)	1386.8	(n = 42)	13.0	(-99.9	;	125.9)	0.82
**Incongruent reaction time** (ms)	1749.3	(n = 436)	1776.4	(n = 42)	27.1	(-110.0	;	164.2)	0.70
**Congruent accuracy** (%)	100.0	(n = 436)	100.0	(n = 42)	0	*			0.55
**Incongruent accuracy** (%)	81.3	(n = 436)	75.0	(n = 42)	-6.3	*			0.27

For inclusion in the "Followed up" group participants needed full data for the main outcome "Math score"

Difference (95% CI) between groups are presented.

* Denotes non-normal distributed data with medians and crude difference presented.

At baseline, the control group had significantly higher PA level (count/min) in math lessons compared with the intervention group (p<0001). Over the course of the intervention, there was a tendency that children in the intervention group increased their physical activity compared to control (Table 3). Yet, these differences were non significant; the difference in change in activity during math was 120.4 counts/min (95% CI -9.0; 249.8, p = 0.07), in favor of the intervention group. Accelerometer wear time and number of days with assessment were not different at baseline between groups (Table 1). During the course of the intervention wear time and number of days of assessment was 13.2 (SD 0.06) hours/day 7.0 (SD 0.2) days and 13.1 (SD 0.04) hours/day and 7.3 (0.1) days in the control and intervention group respectively.

**Table 3:** Difference at follow-up between intervention and control in selected physical activity outcomes—adjusted for baseline level of the outcome, sex and school as random effect. Mixed effect model coefficients.

	Physical activity in Math class	Total physical activity	Total time at MVPA
***Fixed effect***	counts/min (SE)	counts/min (SE)	min/day^a^ (SE)
**Intervention group** ***Active Math vs control (ref*.*)***	120.4 (63.4)	27.0 (28.0)	3.4 (3.1)
Gender *Girls vs*. *boys (ref*.*)*	-66.9 (30.1) *	-33.7 (19.2)	-8.1 (2.2) ***
Baseline level of outcome	0.2 (0.07)**	0.5 (0.06) ***	0.6 (0.05) ***
***Random effects (variance)***			
Intercept Schools	13408.9 (5867.4)	1420.0 (1059.1)	17.8 (12.8)
Residual	82242.7 (5894.1)	24962.4 (2036.4)	292.7 (24.0)
***N***			
Number of observations	398	314	314
Number of schools	12	12	12

* p<0.05

**p<0.01

***p<0.001

^a^ extrapolated to a 14 hours day length

SE, standard error

MVPA, moderate and vigorous physical activity

Math skills were significantly affected by the 9-month active math intervention (Table 4). While the mean score on the math skill test improved in both groups, the intervention group improved math score by 1.2 (95% CI 0.4; 1.9) points more than the control (p = 0.002). Expressed in percentage terms, the control group improved math score by 17.5% and the intervention group by 24.7%, corresponding to a 39% larger improvement in the intervention group. Relative Cohen’s d effect size for group differences in the change in math scores was d = 0.38, whereas absolute effect-sizes were d = 0.91 and d = 1.48 for control and intervention groups, respectively.

**Table 4:** Difference at follow-up between intervention and control in primary and secondary outcomes—adjusted for baseline level of the outcome, sex and school as random effect. Mixed effect model coefficients.

	Primary outcome	Secondary outcomes			
	Mathematics score	Body mass index	Fitness	Incongruent reaction time	Incongruent accuracy
***Fixed effect***	points (SE)	kg/m^2^ (SE)	m (SE)	ms (SE)	% (SE)
**Intervention group** ***Active math vs control (ref*.*)***	1.2 (0.4)*	-0.2 (0.1)	10.0 (13.9)	-8.6 (41.1)	0.7 (1.6)
Gender *Girls vs*. *boys (ref*.*)*	0.5 (0.2)*	0.2 (0.1) *	-31.8 (7.5)***	61.8 (29.1)*	0.3 (1.2)
Baseline level of outcome	0.2 (0.03)***	1.0 (0.02) ***	0.5 (0.04)***	0.3 (0.03)***	0.2 (0.04)***
***Random effects (variance)***					
Intercept Schools	0.5 (0.3)	0.04 (0.03)	527.3 (325.6)	2529.4 (1698.4)	2.6 (3.1)
Residual	5.3 (0.4)	0.5 (0.03)	5359.1 (375.4)	89407.9 (6077.3)	155.7 (10.6)
***N***					
Number of observations	450	461	423	446	446
Number of schools	12	12	12	12	12

*p<0.05

**p<0.01

***p<0.001

SE, standard error

Regarding the secondary outcomes, the Flanker test, BMI and aerobic fitness showed no significant change over time in either group.

**Fig 2:**
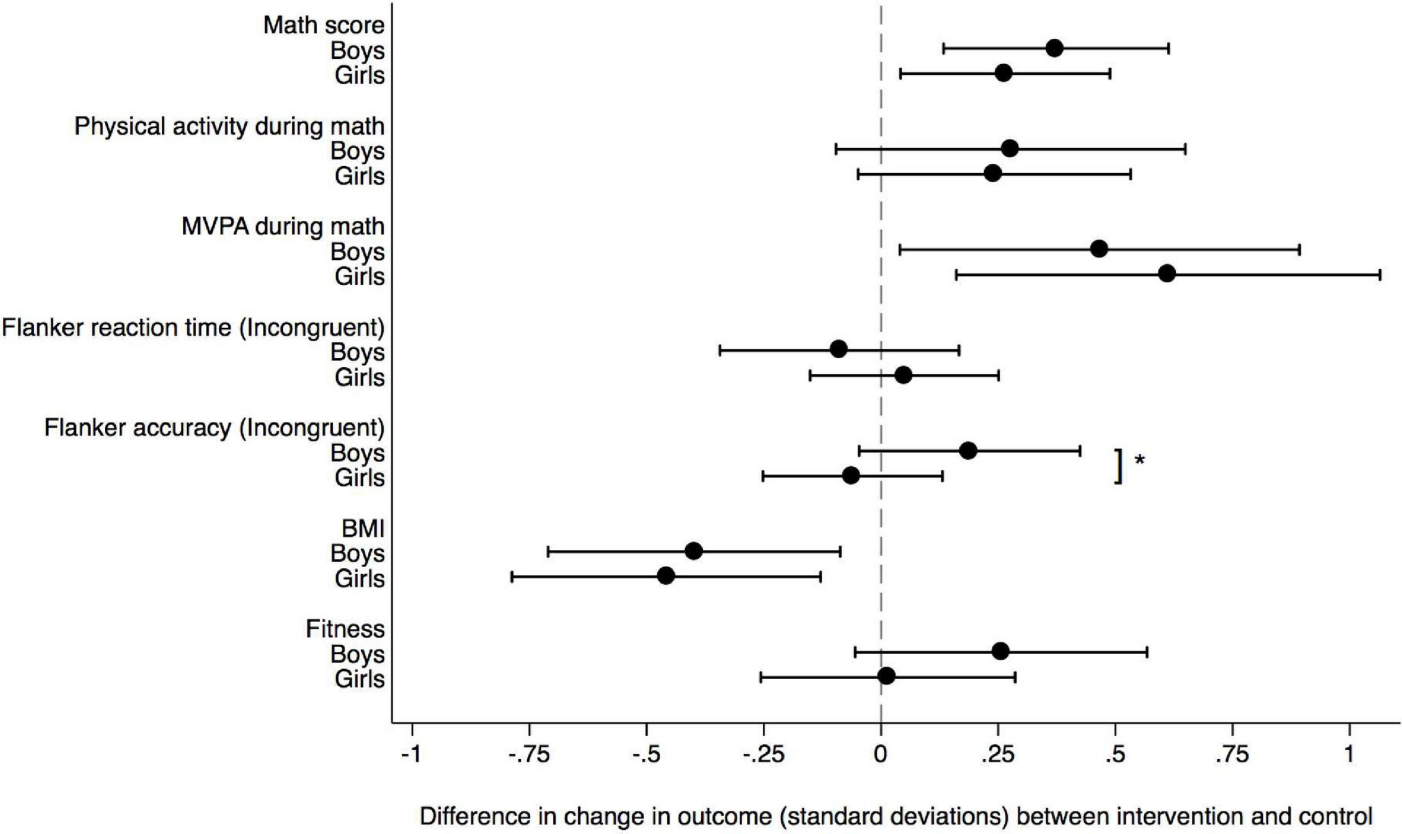
Sex-specific changes in outcomes. Sex-specific changes in outcomes in the intervention group relative to control (= 0) expressed in standard deviations from baseline to 9 months follow up. Mixed models estimates with school class as random effects, including baseline levels as covariates. Significant gender interaction: * p = 0.03.

While the gender intervention interaction term was insignificant for most outcomes, it was significant for the Flanker test, where boys showed greater improvement in accuracy of incongruent trials (p = 0.03) (Fig 2).

Sensitivity analysis revealed no meaningful effect of dropouts on the results.

### General versus task-relevant PA

**Fig 3:**
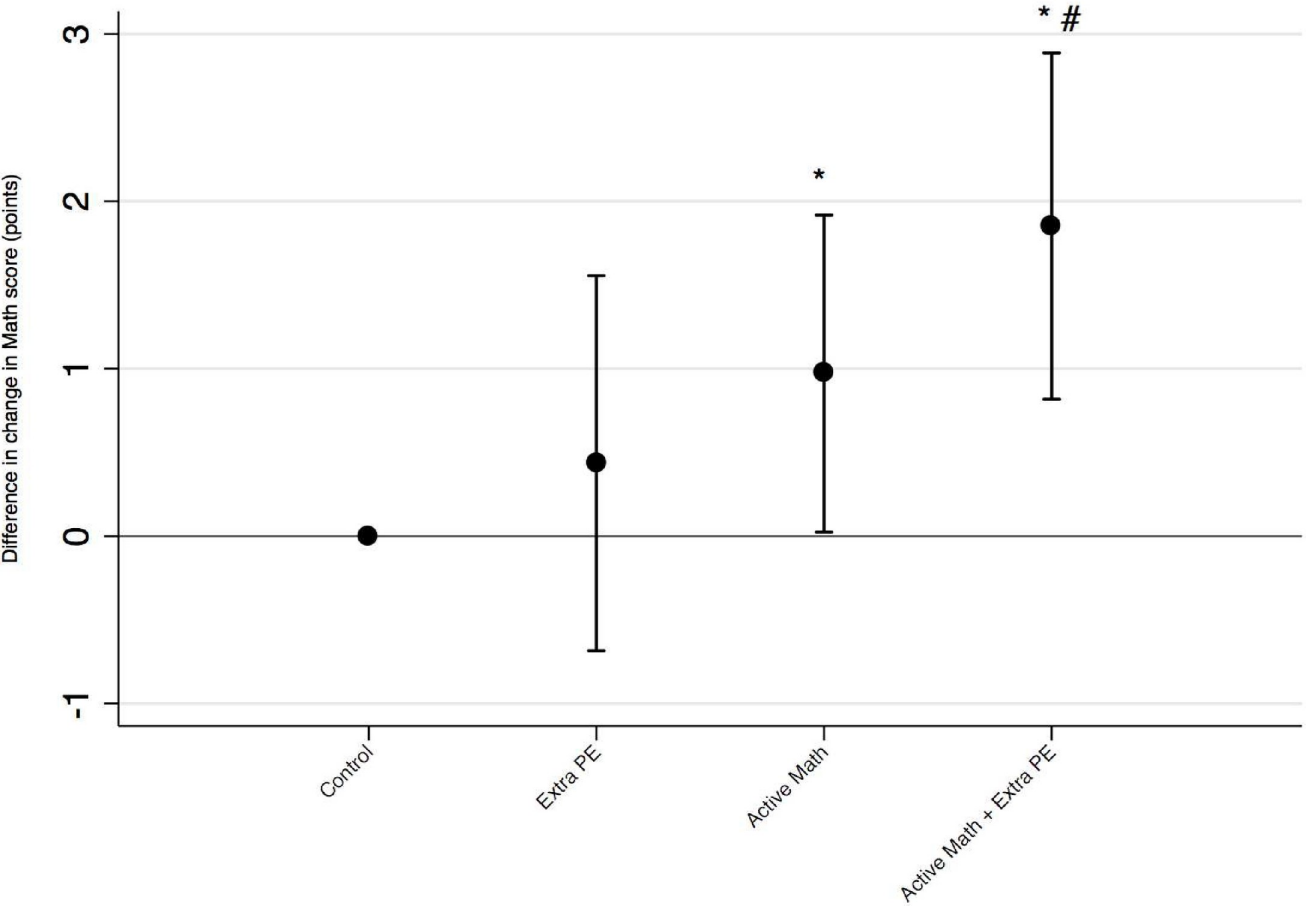
Effect of task-relevant versus general Physical activity. Math score difference in change from control (normal math, 90 min. PE/wk) for subgroups. **Extra PE**: normal math, 270 min. PE/wk, **Active Math**: active math, 90 min. PE/wk, **Active Math + Extra PE**: active math, 270 min. PE/wk. Significantly different from Control: * p<0.05, Extra PE: # p<0.05. Data shown as differences in means.

Post-hoc analysis revealed no significant difference in change in math score between the control group and group with extra physical education classes (Fig 3). The active math intervention improved math score significantly more compared to the controls, and the addition of extra physical education classes to active math increased the positive effect even more. However, this analysis lacks statistical power and randomization.

## Discussion

This large, cluster-randomized controlled trial has shown that classroom-based task-relevant physical active math teaching resulted in a greater improvement of math skills compared to regular math teaching over one school year in 7-year-old schoolchildren. These findings are in line with previous longitudinal studies [13, 25]. Adding an extra 180 minutes of PE did not detract from the children’s math achievement. However, novel teaching strategies incorporating PA appear to provide even greater benefits. We saw no significant effects of increased physical activity during math lessons on executive functions or aerobic fitness. The relative effect-size of d = 0.38 is similar to previous interventions [13].

Two main approaches exist in the literature on the effect of PA on academic achievement [26]. One approach is to investigate *task-irrelevant* whole-body movements that are not integrated into the learning task, with emphasis on the association between health-related variables (such as fitness level, length and intensity of PA) and improvements in brain function and cognitive performance leading to improved academic achievement, e.g. [10]. The other approach is to investigate *task-relevant* part-body movements (most often gestures) that are integrated into the learning task, based on the idea that important cognitive functions are grounded in action and perception as a function of bodily experience and meaningful sensory–motor interactions, e.g. [27]. We attempted to combine these two approaches by integrating task-relevant physical activity into the classroom combined with at least 15 minutes PA per 45 minutes lesson.

The improvement in math skills from increased PA levels during math lessons could have several explanations such as physiological adaptations, the task-relevant character of the PA, and/or the type of teaching in the active math intervention. Physiological adaptations due to immediate and regular PA include changes in brain blood flow and arousal level, improved conduction of information, increased oxygen levels to areas of the brain that support memory, and increased grey and white matter [28, 29]. Whether these changes have occurred in this intervention is uncertain, as these adaptations have mostly been associated with moderate to vigorous PA [30]. However, while there was no required intensity of the classroom-based physical activity in the present study, the intervention group there was a tendency that children in the intervention group increased their physical activity compared to the controls.

A critical factor affecting math skills may also have been the task-relevant and cognitively engaging character of the PA applied in the active math intervention, with components from the theory of embodied cognition such as enriched encoding (coupling of bodily experience to abstract information) [31, 32] and effectiveness of working memory subsystems (distribution of cognitive load on several working memory subsystems) [33]. The task-relevant activities provided a supplementary physical stimulus to the visual and auditory stimuli connected to learning of abstract math topics, thus offering additional memory cues. This activation of multiple areas of the brain cortex during task-relevant PA may have prevented overload of the working memory, as suggested in a previous study where part-body movements (gestures) possibly freed up working memory resources for use in deeper understanding [34, 35].

A third possible explanation for our findings of improved math skills may simply be that the nature of the active math teaching was different to traditional teaching, and the children were thus more motivated [36]. The limited post-hoc analysis argues against this, however, as the results for active math should then have been similar to active math and extra physical education classes. Larger studies would be needed to further investigate this aspect.

The present study showed no significant effect of PA on the modified Flanker task. This contrasts with previous studies [8–11] and may be due to the low number of tasks used in our study compared to other studies i.e. 17 and 45 vs. 100 [7]. Our results may thus not be an accurate representation of the response, especially given the great variability observed in youth [37]. The absence of intensity requirement and the possibility of PA durations as short as 15 minutes per lesson could also explain the absence of executive function adaptations, as positive correlations have been shown with moderate to vigorous physical activity and longer duration e.g. [8, 10]. However, PA emphasizing cognitive effort and mental engagement has previously improved executive functions [38, 39], and we expected that our task-relevant and cognitive challenging PA would also be associated with an improvement. The finding that active math appeared to be beneficial for boys’ flanker performance could be linked to studies indicating that brain connections tend to optimize earlier in girls, maybe resulting in faster cognitive maturity in specific cognitive aspects compared to boys [40]. However, sex differences in cognition are rarely investigated and when they are, they seldom supply any notable conclusions [41].

Our finding that classroom-based physical activity did not reduce BMI differs from previous findings [25, 42]. However, the inclusion of PA in only one subject (math) compared to PA included across the entire curriculum may be insufficient to trigger an effect on BMI.

Different initiatives were made to ensure compliance with the intervention program and assessment of compliance. Unfortunately, not all data were sufficient to describe compliance appropriately. The accelerometer assessment of PA during math lessons is one measure of compliance, however, this is limited by the very short-term period of assessment. In general retrieval of teacher reports was difficult despite thorough explanation about the importance of the information. In agreement with teachers, reports were initially in paper form to be filled out after a lesson and subsequently distributed via e-mail, but with very low response rates for both methods. Finally, SMS-tracking was introduced, still providing a low response rate caused by limited resources at the schools, e.g. limited time available for teachers during recess. The lack of detailed information on compliance during the 9-month intervention is a threat to the internal validity, however, this is an inherent limitation of pragmatic trials. It would have been valuable to have data on ways the teachers had interacted with the children during math lessons to investigate the complexity of the social contexts in which the active math was learned. Approaches could include case studies of a specific teacher or context, observations of classroom activities, or qualitative interviews with children, teachers, and parents. Another limitation in this study was that we used a new math skill test, thus limiting comparison with previous studies. Finally, although we found a tendency towards increases in PA during math lessons in the intervention group, our objective assessment of PA during math may not be sufficiently reliable due to the relatively low amount of lessons we assessed.

## Conclusion

The findings of this large RCT among 7-year-olds suggest that integration of physical activity into math classes over a school year improved academic achievement in math skills. Furthermore, a combination of classroom-based physical activity and more physical education classes was the most beneficial for math skills.

‘Moving classrooms’ may be an effective approach to improve academic results and general health in primary school. We need further research into how type, quality, intensity, and duration of physical activity influence the learning outcome, and would need to control for aspects such as teacher motivation and a novelty effect of a new teaching method. A multifactorial randomized design would allow comparison of different intervention types.

## Supporting information

S1 Fig (PDF)

S1 File (PDF)

S2 File (PDF)

## Abbreviations

PA: Physical activity
SMS: Short message service
PE: Physical education
MVPA: moderate-to-vigorous physical activity
