# Supplementary material for: Classroom-based physical activity improves children’s math achievement – A randomized controlled trial
Source: PLoS One. 2018 Dec 17;13(12):e0208787. doi: 10.1371/journal.pone.0208787 (PMC6296522; doi:10.1371/journal.pone.0208787)
Supplement: S1 File — (PDF) [file pone.0208787.s002.pdf]

S1 File. Approval from the Scientific Ethical Committee. Translation of relevant parts of the project approval from the scientific committee of the Region of Southern Denmark

To clinical professor and chief physician Niels Wedderkopp

Department of Sports Science and Clinical Biomechanics

University of Southern Denmark

Campusvej 55, DK-5230 Odense M

Regarding the research project: The effect of increased physical activity on present and future health in children and adolescents. An intervention project over 3 years in the municipality of Svendborg.

The scientific ethical committee of the Region of Southern Denmark has the 10th of November 2010 received an additional entry with entry no. 27748 dated the 8th of November 2010.

Addition no. 4 regards:

The effect of increased physical activity on children's learning ability, a new partial study under the Svendborg project:

The ongoing Svendborg project provide a unique possibility to investigate the influence of physical activity on children's cognitive abilities. The purpose with this additional study is thus to investigate the connection between children's physical activity and cognitive abilities; partly via the existing implementation of 6 hours physical education per week which occurs regardless of the academic subjects, and partly via physical activity in the mathematics teaching.

The committee has no remarks to the submitted material and can approve the addition above.

The approval includes the following documents:

- Signed entry of additional entry dated 8th of November 2010
- Participant information received 10th of November 2010

10 March 2016

Endorsed by

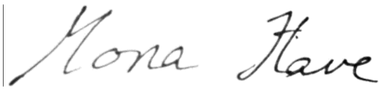

Mona Have
PhD Student, Department of Sports Science and Clinical Biomechanics
Tel. +45 6550 3441
Email mhsorensen@health.sdu.dk
Addr. Campusvej 55, DK-5230 Odense M, Denmark
