# Supplementary material for: Classroom-based physical activity improves children’s math achievement – A randomized controlled trial
Source: PLoS One. 2018 Dec 17;13(12):e0208787. doi: 10.1371/journal.pone.0208787 (PMC6296522; doi:10.1371/journal.pone.0208787)
Supplement: S2 File — (PDF) [file pone.0208787.s003.pdf]

S2 File. Approval from the Scientific Ethical Committee. Original project approval from the scientific committee of the Region of Southern Denmark

Den Videnskabsetiske Komité
for Region Syddanmark

komite@regionsyddanmark.dk

Klinisk professor, overlæge
Niels Wedderkopp
Østrupvej 18
5210 Odense NV

25. november 2010

Projekt-ID: S-20080047
CKH/csf

Vedr. forskningsprojekt: Betydningen af øget fysisk aktivitet for nuværende og fremtidig sundhed hos børn og unge. Et interventionsprojekt over tre år i Svendborg kommune.

Den Videnskabsetiske Komité for Region Syddanmark har den 10. november 2010 modtaget en anmeldelse af tillæg med anmeldelses nr. 27748 dateret den 8. november 2010.

Tillæg nr. 4 omhandler:
Betydningen af øget fysisk aktivitet for børns indlæringsevne et nyt delstudie i Svendborgprojektet:
Det igangværende Svendborg-projekt giver en unik mulighed for at undersøge fysisk aktivitets indflydelse på børns kognitive evner. Formålet med dette tillægsstudie er således at undersøge sammenhængen mellem børns fysiske aktivitet og kognitive evner; dels via den eksisterende implementering af 6 timers idrætsundervisning i ugen, som foregår uafhængigt af de akademiske fag, og dels via fysisk aktivitet i matematikundervisning. 4 af de allerede igangværende forsøgsskoler inkluderes i den kommende supplerende intervention, som foregår 15 måneder med elever i indskolingen. De fire grupper i interventionen ser ud som følger: Forsøgsgruppe 1: På en idrætsskole, hvor eleverne i forvejen har 6 timers fysisk aktivitet via bl.a. ekstra idrætstimer og morgenaktiviteter, vil 3 klasser samtidig modtage fysisk aktivitet i deres matematikundervisning, for at teste hvorvidt en eventuel kognitiv effekt kun indfinder sig, når den øgede fysiske aktivitet både finder sted i og udenfor undervisningen. Forsøgsgruppe 2: På en kontrolskole, hvor eleverne ikke har ekstra fysisk aktivitet, vil 3 klasser modtage fysisk aktivitet i deres matematikundervisning, således at det kan testes, hvorvidt den øgede fysiske aktivitet i undervisningen har afgørende effekt på kognitive evner. Kontrolgruppe 1: Består af 3 klasser fra en idrætsskole, som har øget fysisk aktivitet udenfor undervisning for at teste, i hvilken grad øget fysisk aktivitet generelt skaber effekt på indlæringen, og hvorvidt en eventuel effekt er forskellig fra effekten af fysisk aktivitet i undervisning. Kontrolgruppe 2: Består af 3 klasser fra en kontrolskole, som hverken har ekstra fysisk aktivitet i eller udenfor undervisning for at teste, om den kognitive effekt af dette fysiske aktivitetsniveau er anderledes end hos de andre grupper.

Komiteen har ingen bemærkninger til det fremsendte materiale og kan godkende ovenstående tillæg.

Godkendelsen omfatter følgende dokumenter:

- Underskrevet anmeldelse af tillæg dateret den 8. november 2010
- Deltagerinformation modtaget 10. november 2010

Sagen har været behandlet og godkendt af Komiteens formand, overlæge, dr.med. Birger Møller den 24. november 2010.

Behandling af forskningsprojekter og tillægsprotokoller i de videnskabsetiske komitéer er som udgangspunkt gebyrbelagt. Der betales dog ikke gebyr for projekter og tillægsprotokoller, der indleveres til den regionale videnskabsetiske komite af institutioner, som komiteens egen region afholder udgifterne til. For øvrige projekter, der indleveres fra f.eks. statsinstitutioner, private hospitaler og firmaer, betales et gebyr pr. projekt eller tillægsprotokol. Ligeledes betales gebyr for projekter, der indleveres fra en regional institution i samarbejde med f.eks. statsinstitutioner, private hospitaler, fonde og firmaer. Komitéen skal derfor anmode om gebyrbetaling kr. 1. 500,- for behandling af ovennævnte tillæg. Beløbet bedes venligst indbetalt på bankkonto i **Spar Bank Nord, Reg.-nr. 9035 Kontonr. 457 1868 968. Indbetalingen, bedes mærket Videnskabsetisk Komite samt anmeldelses nr. eller den forsøgsansvarliges navn.**

Venlig hilsen

Claus Kvist Hansen
Sekretariatsleder

Kopi til: Cand. Mag., Mona Have Sørensen, Allegade 20 A, 5000 Odense C.
